# Assessing the Impact of Multi-Morbidity and Related Constructs on Patient Reported Safety in Primary Care: Generalized Structural Equation Modelling of Observational Data

**DOI:** 10.3390/jcm10081782

**Published:** 2021-04-20

**Authors:** Ignacio Ricci-Cabello, Aina María Yañez-Juan, Maria A. Fiol-deRoque, Alfonso Leiva, Joan Llobera Canaves, Fabrice B. R. Parmentier, Jose M. Valderas

**Affiliations:** 1 Health Research Institute of the Balearic Islands (IdISBa), Carretera de Valldemossa, 79 Hospital Universitario Son Espases, Edificio S, 07120 Palma, Spain; ignacio.ricci@ssib.es (I.R.-C.); aina.yanez@uib.es (A.M.Y.-J.); mariaantonia.fiol@ssib.es (M.A.F.-d.); aleiva@ibsalut.caib.es (A.L.); jllobera@ibsalut.caib.es (J.L.C.); fabrice.parmentier@uib.es (F.B.R.P.); 2 Balearic Islands Health Services, Primary Care Research Unit of Mallorca, 07002 Palma, Spain; 3 CIBER de Epidemiología y Salud Pública (CIBERESP), 28029 Madrid, Spain; 4 Department of Nursing and Physiotherapy and Global Health Research Group, University of the Balearic Islands, 07122 Palma, Spain; 5 Primary Care Prevention and Health Promotion Research Network, RedIAPP, 28029 Madrid, Spain; 6 Department of Psychology and Institute of Health Sciences (iUNICS), University of the Balearic Islands, 07122 Palma, Spain; 7 School of Psychology, The University of Western Australia, Perth, WA 6009, Australia; 8 Health Services & Policy Research Group, Exeter Collaboration for Academic Primary Care, NIHR School for Primary Care Research, University of Exeter, Exeter EX1 2HZ, UK; 9 NIHR South West Peninsula Applied Research Collaboration, University of Exeter, Exeter EX1 2HZ, UK; 10 Department of Medicine, Yong Loo Lin School of Medicine, National University of Singapore, Singapore 119077, Singapore

**Keywords:** multimorbidity, polypharmacy, patient safety, primary care, structural equation model, latent class analysis

## Abstract

We aimed to examine the complex relationships between patient safety processes and outcomes and multimorbidity using a comprehensive set of constructs: multimorbidity, polypharmacy, discordant comorbidity (diseases not sharing either pathogenesis nor management), morbidity burden and patient complexity. We used cross-sectional data from 4782 patients in 69 primary care centres in Spain. We constructed generalized structural equation models to examine the associations between multimorbidity constructs and patient-reported patient safety (PREOS-PC questionnaire). These associations were modelled through direct and indirect (mediated by increased interactions with healthcare) pathways. For women, a consistent association between higher levels of the multimorbidity constructs and lower levels of patient safety was observed via either pathway. The findings for men replicated these observations for polypharmacy, morbidity burden and patient complexity via indirect pathways. However, direct pathways showed unexpected associations between higher levels of multimorbidity and better safety. The consistent association between multimorbidity constructs and worse patient safety among women makes it advisable to target this group for the development of interventions, with particular attention to the role of comorbidity discordance. Further research, particularly qualitative research, is needed for clarifying the complex associations among men.

## 1. Introduction

The safety of primary care is a global priority for healthcare, led by the World Health Organization [1]. Patient safety is defined as “the avoidance, prevention and amelioration of adverse outcomes or injuries stemming from the process of healthcare” [2], and the harm arising from a patient safety incident as “impairment of structure or function of the body and/or any deleterious effect arising there from, including disease, injury, suffering, disability and death, and may be physical, social or psychological” [1]. Around 2% to 3% of all primary care encounters result in a patient safety incident, with 1 in 25 causing a serious harm outcomes [3]. These incidents are frequently related to diagnosis (either delayed or missed) or to treatment (delayed or inappropriate) [4,5]. A number of different factors contribute to these incidents, such as the working environment, information transfer at the primary–secondary interface, doctor–patient relationship or continuing education [6,7,8]. 

Multimorbidity, the presence of more than one health condition in an individual [9], is increasingly prevalent and represents a major part of the workload of primary care [10,11]. Although the prevalence increases substantially with age, in absolute terms, multimorbidity is more prevalent in those aged 65 years or less and is much more common in socioeconomically deprived areas, so that they develop equivalent levels of multimorbidity 10–15 years before their more affluent peers [12]. It challenges the usual care delivery, which is frequently structured around pathways of care for single diseases [13,14]. This can generate a tension between applying single-condition guidelines to patients with multimorbidity as security against uncertainty or penalty and potentially causing patients harm [15,16]. People suffering from multiple health conditions are more likely to require an increased number of healthcare processes, triggering the involvement of an increased number of health professionals [13,14,17]. This increased complexity in the delivery of care threatens coordination and continuity, thereby decreasing the likelihood of receiving care that meets the appropriate standards for patient safety [14]. 

A previous systematic review of the relationship between multimorbidity and patient safety incidents in primary care found that both mental–physical multimorbidity and physical multimorbidity were associated with a higher risk for active safety incidents (such as adverse drug events and medical complications), whereas mental–physical comorbidity (mainly depression) was associated with an increased risk for both active safety incidents and precursors of safety incidents (such as a lower quality of care, prescription errors and medication nonadherence) [18]. In developed countries, approximately 30% of patients aged 65 years or older are prescribed five or more drugs. Whereas many may benefit from such polypharmacy [19], it comes with an increased risk of adverse events in older people due to the physiological changes of aging that alter the pharmacokinetic and pharmacodynamic responses to drugs [20]. In addition to polypharmacy, other important sources of unsafe care among patients with multimorbidity are communication-related incidents, e.g., incomplete or nontransfer of information across care boundaries and clinical decision-making incidents that lead to the most serious patient harm outcomes. 

However, multimorbidity might be associated with higher (rather than lower) levels of patient safety if the intrinsic high-risk profile of these patients results in an increased patient safety activation of either the patients or the health professionals involved or both. The presence of synergistic combinations may also provide increased incentives for both patients and professionals for adhering to the guidelines for recommended care [21]. Indeed, in some cases, multimorbidity has been associated with a lower risk for safety failures (e.g., a trend was observed for physical multimorbidity to be associated with better quality of care) [18].

The relationship between multimorbidity and patient safety outcomes in primary care is complex, with high levels of variability, and is influenced by differences in how multimorbidity is measured [18]. Attempts to study the impact of multimorbidity are complicated by the lack of consensus about how to measure the concept [13]. Related constructs, such as comorbidity (sets of conditions that coexist with a predefined index condition, further classified as concordant and discordant based on the pathophysiology [22,23]; morbidity burden (total burden of physiological dysfunction, which is affected both by the number and the severity of conditions [9,24]) and clinical complexity (which acknowledges that the complexity of care provision for any individual is influenced not only by health-related characteristics but, also, by the socioeconomic, cultural, environmental and patient behaviour characteristics [9,25]) are often used interchangeably [26]. Studies simultaneously using and comparing the multiple approaches to measure multimorbidity are very much needed to shed light on the mechanisms by which multimorbidity affects patient safety and to identify groups of multimorbid patients at higher risk of safety events. 

The aim of this study was to examine the complex relationships between patient-reported patient safety outcomes and multimorbidity as conceptualized and measured using the constructs of multimorbidity and polypharmacy, comorbidity, morbidity burden and patient complexity and the pathways by which the multimorbidity constructs may be associated with patient-reported patient safety in primary care.

## 2. Materials and Methods

### 2.1. Design

This is a cross-sectional study combining baseline data from the SinergiAPS phase II and phase III trials [27]. The SinergiAPS project aims to develop and evaluate the effectiveness of an intervention to improve patient safety in PHC centres by providing them with patient feedback obtained through the administration of a standardized self-reports of patient safety. Details of the phase II study are available elsewhere. In brief, the phase II study consisted in a 3-month, uncontrolled, pre-post-study, including 10 primary healthcare (PHC) centres in Mallorca, Spain. Baseline data collection took place between October and November 2018. In each PHC centre, all patients in the waiting room were consecutively approached by a research assistant and invited to complete the Spanish version of the Patient Reported Experiences and Outcomes of Safety in Primary Care (PREOS-PC) questionnaire to report on their perceptions, experiences and outcomes in relation to the safety of the healthcare received from their PHC centre over the previous 12 months. Sociodemographic and clinical information was also collected. All patients having visited their PHC centre at least once during the previous 12 months were considered eligible to complete the questionnaire. Patients aged <18 were eligible only if they were accompanied by a carer or family member aged 18 or more willing to complete the questionnaire on their behalf. Questionnaires were primarily self-administered using table computers. However, paper versions of the questionnaire were also available upon request. Patients were also given the opportunity of having the questionnaire administered by the researchers in situ. We aimed to collect 50 questionnaires per centre. Questionnaire completion time was, on average, 15 min. The phase III consisted in a 12-month, two-arm, two-level cluster randomized controlled trial, which is currently ongoing. It involves 59 PHC centres in Mallorca (30 centres) and Catalonia (29 centres) that were selected to ensure variations in terms of the list size, rurality and deprivation. Baseline data collection took place between June 2019 and January 2020 and involved the administration of 75 patient questionnaires per centre (4425 questionnaires in total), following the same methodology previously described for the phase II trial.

### 2.2. Multimorbidity and Related Constructs

**Figure 1:**
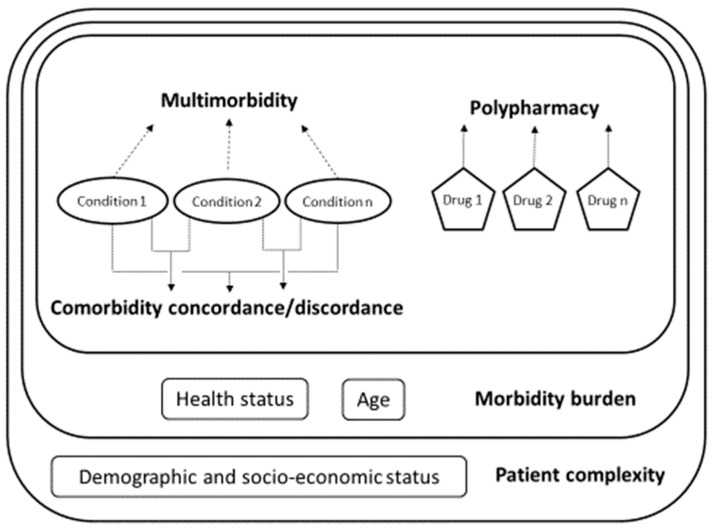
Multimorbidity and related constructs.

We enhanced the proposal of Valderas et al. [9], which distinguished four key comorbidity constructs: multimorbidity, comorbidity, morbidity burden and patient complexity, with the addition of polypharmacy (Figure 1).

1. Multimorbidity and polypharmacy. We measured multimorbidity as the total number of self-reported long-term conditions for each respondent [28] (see below for the full list). Previous studies measuring self-reported multimorbidity support the validity of the self-reported approach [28,29,30,31]. Polypharmacy estimates were based on the self-reported number of prescription drugs.
2. Comorbidity discordance. We classified pairs of conditions as concordant or discordant based on their pathophysiology. Concordant comorbidity was defined as a set of conditions that are part of a shared pathophysiological pathway and thereby more likely to share the same management and are more likely to be the focus of the same disease management plan (e.g., hypertension and diabetes) [22,23]. Discordant comorbidity was defined as sets of diseases that are “not directly related in either pathogenesis or management and do not share an underlying predisposing factor” (e.g., hypertension and osteoporosis). We hence classified as concordant comorbidities the following sets of conditions: cardiovascular (which included “hypertension”, “hypercholesterolemia”, “type 2 diabetes”, “long-term heart problem” and “blood circulation problems”); mental health (“depression” and “other mental health problems”) and musculoskeletal (“arthrosis and rheumatic problems” and “osteoporosis”). The rest of the conditions, including “asthma or bronchitis or emphysema”, “allergy”, “migraine or headaches”, “prostate-related problems”, “peptic or gastric ulcer”, “inguinal hernia” and “menstruation-related problems”, were not considered to be concordant with any other according to their pathophysiology. All patients with more than one condition were thus classified in terms of increasing the levels of comorbidity discordance as having (mutually exclusive, lowest-to-highest discordance): (1) fully concordant multimorbidity (100% of the conditions classified as concordant) or (2) predominantly concordant multimorbidity (at least one discordant condition and >50% of the conditions classified as concordant), predominantly discordant multimorbidity (at least one discordant condition and ≤50% of the conditions classified as concordant) and totally discordant multimorbidity (100% of conditions mutually discordant).
3. Morbidity burden. We developed an index of morbidity burdens based on the previous constructs (multimorbidity (number of conditions), polypharmacy (number of medications and comorbidity discordance), and self-reported health status and age (see details in Statistical Analysis).
4. Patient complexity. Finally, we constructed an index of patient complexity based on the variables included in the development of the morbidity burden and included the educational attainment, occupational status and country of origin (see details in Statistical Analysis).

### 2.3. Patient Safety

Patient safety was measured with the Patient Reported Experiences and Outcomes of Safety in Primary Care (PREOS-PC) questionnaire [32]. PREOS-PC invites patients to report on their perceptions and experiences concerning the safety of healthcare in their practice over the past 12 months. In this study, we used the validated PREOS-PC 27 items Spanish version, which covers five main domains: practice activation (what does the practice do to create a safe environment and to ensure safety). patient activation (how pro-active are patients in ensuring safer healthcare), experiences of patient safety events (errors). outcomes of patient safety (harm) and patients’ overall perception of safety (how safe do patients think the practice is) [33]. 

Scale scores were calculated as the percentage of the maximum score achievable on all items, with scores ranging from 0 to 100 (higher scores correspond to higher levels of patient safety). For multi-item scales, a scale score was derived using the available items without any imputation, except when more than 50% of the items were missing (scale scored as missing). We developed an overall score of Patient Safety based on PREOS-PC scales scores using a latent class analysis (see Statistical Analysis for details). 

Further details on the conceptual framework and the development process, validation and psychometric properties of the self-reported measures PREOS-PC survey are available elsewhere [32,33,34].

### 2.4. Statistical Analysis

An assessment of model identification was made using the two-step rule [35]. First, we used separate confirmatory factory analyses to develop the latent variables corresponding to the domains “patient safety”, “morbidity burden” and “patient complexity” based on the set of predefined observed variables previously described (see above). The suitability of the allocation of each indicator to its corresponding domain was examined based on their correlations and the loadings after a confirmatory factor analysis. We also estimated a goodness of fit for the three models through estimation of the Standardized Root Mean Squared Residual (SRMR) [36], Comparative Fit Index [37], Root Mean Square Error of Approximation (RMSEA) and equation-level goodness of fit.

The association between the multimorbidity measures, number of visits and patient safety was initially examined using separate bivariate and adjusted lineal regression models for each multimorbidity construct as the sole independent variable and the patient safety latent variable as the dependent variable. We then constructed generalized structural equation models (GSEM) [38,39]. GSEM allows to evaluate simultaneous relationships among variables and is also useful to assess models with categorical variables and unobservable latent variables, since a GSEM structure links are latent and its measurements variable. We used separate GSEM models for each of the multimorbidity and related constructs. For the model on comorbidity, discordance estimates were calculated using the completely concordant comorbidity group as the reference group. A statistical analysis comprised the estimation of nonstandardized coefficients and 95% CI. Akaike’s information criterion (AIC), which provides a trade between the goodness of fit and model simplicity, was calculated for all GSEM models to facilitate a comparison and identification of the best-performing model [40,41]. Given the exploratory, rather than confirmatory, nature of our analysis, it was deemed a more suitable than the alternative Bayesian information criterion [42]. All analyses were stratified by gender, and all regression and GSEM analyses were adjusted by age, educational attainment and self-reported health status (except in those cases where these potentially confounding variables were already used for the construction of our multimorbidity latent measures). We were not able to use multilevel GSEM because of the computational limitations. We therefore conducted sensitivity analyses using multilevel regression models to adjust for clustering effects (as our Patient Safety measure was likely to cluster among practices). 

**Figure 2:**
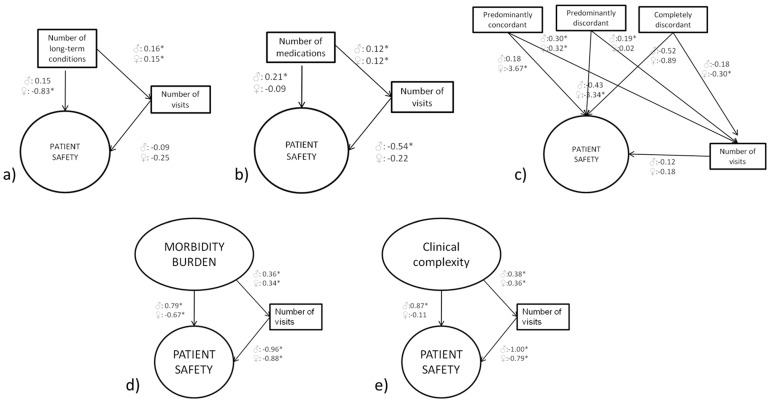
Association between multimorbidity and related constructs and patient safety. (**a**) Association between the number of conditions, patient safety and number of visits in men and women. (**b**) Association between the number of medications, patient safety and number of visits in men and women. (**c**) Association between level of discordance among conditions, patient safety and number of visits in men and women. (**d**) Association between morbidity burden, patient safety and number of visits in men and women. (**e**) Association between the clinical complexity, patient safety and number of visits in men and women. Statistically significant associations (*p* < 0.05) are noted with an asterisk (*).

We hypothesized that multimorbidity constructs would be associated with patient safety both via a direct and indirect pathway (Figure 2). Whereas, through the direct pathway, we hypothesized a direct association between higher levels of multimorbidity and worse patient safety; through the indirect pathway, we hypothesized that this association would be mediated by the intensity of interactions with General Practice (i.e., higher multimorbidity directly associated with a higher number of visits and a higher number of visits associated with worse patient safety). Increased number visits may both offer increased opportunities to addressing patient safety issues and, also, to increase the potential for additional patient safety issues to arise. We did not make any a priori hypothesis as to what of these potential mechanisms would be a larger impact on the net effect in the indirect pathway. We hypothesized an inverse association between multimorbidity constructs and patient safety via a direct pathway (higher levels of multimorbidity and related constructs associated with higher levels of health care use and the latter associated with lower levels of patient safety). 

Questionnaires with missing data were excluded from the analyses. We used Stata v15.1 and applied an α level of 5% throughout. 

## 3. Results

Of the 6393 patients invited, 5010 accepted to complete the questionnaire (response rate = 78.4%). After excluding 228 questionnaires with incomplete data, the baseline data were available for 4782 adult patients from 69 primary care centres (Table 1). About two-thirds of respondents were women, mean age was 52, half of them were working and a third were retired. Almost half (44%) had visited their primary healthcare centres more than five times during the last 12 months, and almost 80% had been registered at their centre for more than five years. The population was broadly similar to that of other PHC centres in the region. There were small but statistically significant differences between women and men in terms of age, health status, educational and occupational status and migration status (*p* < 0.05).

The most prevalent chronic conditions for both men and women were hypertension, arthrosis and rheumatic problems and hypercholesterolemia. Women reported more frequently than men osteoporosis, migraines, depression and arthrosis and rheumatic Problems, and about 8% of them reported period-related problems. Men reported more frequently diabetes and heart problems, and 17% reported prostate-related problems.

### 3.1. Multimorbidity and Related Constructs

More than half of the women (53%) presented with multiple long-term conditions, with an average of 2.2 conditions (men: 55%; 2.1 conditions; both comparisons nonsignificant (n.s.)). Approximately half of the women (48%) were taking multiple medications, with an average of 2.3 medications (men: 53% (n.s.); 2.6 (*p* < 0.01)). A small proportion of both women and men reported taking more than 10 medications (2%).

Of the 2216 patients with more than one condition, 346 (13%) patients were classified as having fully concordant multimorbidity, 283 (11%) as predominantly concordant multimorbidity, 1291 (50%) as having predominantly discordant multimorbidity and 646 (25%) as totally discordant multimorbidity. There were significant differences between men and women (*p* < 0.01), with four out of five women (vs. two out three men) having predominantly or completely discordant conditions and one out of 10 women (vs. one out five men) having completely concordant conditions.

#### 3.1.1. Morbidity Burden

Loadings for the latent variable “morbidity burden” (Appendix A, Table A1) ranged from 0.38 to 0.98, whereas the correlations ranged from 0.38 to 0.99 (all statistically significant). The Standardized Root Mean Square Residual (0.04) and Coefficient of Determination (0.97) suggested an adequate model fit for the latent variable “morbidity burden”.

#### 3.1.2. Patient Complexity

Loadings for the latent variable “patient complexity” (Appendix A, Table A2) ranged from 0.12 to 0.97, whereas correlations ranged from −0.24 to 0.99 (all statistically significant). The Standardized Root Mean Square Residual (0.06) and Coefficient of Determination (0.96) suggested an adequate model fit.

### 3.2. Patient Safety

Mean scores for the PREOS-PC scales were high (>80/100), indicating high levels of patient safety, for all the scales except for the scale patient activation (Table 2). No clinically relevant differences were observed between men and women in any of the scale scores.

In the confirmatory factor analysis, the latent variable was initially proposed based on all the six PREOS-PC scales (Appendix A, Table A3). All scales loadings (>0.45) supported the validity of the latent variable, with the exception of “Patient Activation” (loading = 0.32). After removing this scale, the loadings ranged from 0.46 to 0.79, suggesting that all the scales explained a significant proportion of the variance of “Patient Safety”. All the correlations between the individual PREOS-PC scales and the “Patient Safety” were statistically significant (*p* < 0.001) and ranged from 0.55 to 0.93. The Standardized Root Mean Square Residual (0.08) and Coefficient of Determination (0.76) suggested an adequate model fit.

### 3.3. Association between Multimorbidity and Related Constructs and Patient Safety

#### 3.3.1. Multimorbidity

In the bivariate lineal regression analysis, the number of conditions was associated in women with lower patient safety, as predicted, but with higher patient safety in men (Online Supplementary Material). The multivariate multilevel lineal regression confirmed the results for women, while the results for men were not significant.

In GSEM (Figure 2a and Table 3), a higher number of conditions in women was associated with worse patient safety through the direct pathway, whereas no statistically significant associations were found through the indirect pathway (i.e., the association between number of conditions and patient safety was not mediated by number of visits, because the number of visits was not associated with patient safety). For men, no significant associations were observed via the direct or indirect pathways.

#### 3.3.2. Polypharmacy

In both the bivariate and the multivariate multilevel lineal regression analyses, the number of medications was not associated with patient safety among women, while (against our hypotheses) a higher number of medications was associated with higher levels of patient safety for men.

The GSEM (Figure 2b) confirmed both the absence of association for women via either pathway and the association with better (rather than worse) patient safety via the direct pathway for men. A simultaneous association with worse patient safety (consistent with a priori hypotheses) was also observed for men.

#### 3.3.3. Comorbidity Discordance

In the bivariate lineal regression analysis, a higher level of discordance was associated with worse patient safety in both women and men, but the associations were rendered not significant in the multivariate multilevel lineal regression analysis.

For women, however, statistically significant associations between patient safety and two of the comorbidity discordance categories (“predominantly concordant” and “predominantly discordant”) were observed in the GSEM analysis via the direct pathways, whereas no significant associations were observed via the indirect pathways (number of visits not associated with patient safety). For men, GSEM confirmed a lack of association via either pathway (Figure 2c).

#### 3.3.4. Morbidity Burden

In both the bivariate and the multivariate multilevel lineal regression analyses, a higher morbidity burden was associated with a lower patient safety in women and, contrary to our expectations, with a higher level of patient safety in men.

In the GSEM analysis (Figure 2d), a higher morbidity burden was associated in women with lower patient safety via both pathways and for men via the direct pathway. At the same time, the inverse association was observed for men via the direct pathway, against the a priori hypotheses and consistent with the observations for a polypharmacy.

#### 3.3.5. Patient Complexity

In both the bivariate and the multivariate multilevel lineal regression analyses, in men, a higher patient complexity was associated with a higher patient safety, while no significant association was observed in women.

In the GSEM analysis (Figure 2e), an association between higher patient complexity and lower patient safety was observed both for men and women via the indirect pathway (as, in both cases, a higher clinical complexity was associated with an increased number of visits, which, in turn, was associated with lower patient safety). For men, a higher patient complexity was directly associated with better patient safety (against our a priori hypotheses), whereas, for women, no significant association was observed between patient complexity and patient safety via the direct pathway.

For women, a consistent association between the lower levels of patient safety and higher levels of the multimorbidity (Figure 2a), discordant comorbidity (Figure 2c), morbidity burden (Figure 2d) and clinical complexity (Figure 2e) was observed via either the direct pathway, the indirect pathway (i.e., mediated by number of visits during the last 12 months) or both. For men, an association between lower patient safety and higher levels of polypharmacy (Figure 2b), morbidity burden (Figure 2d) and clinical complexity (Figure 2e) was observed only via the indirect pathway. However, unexpected associations between higher patient safety and higher levels polypharmacy, morbidity burden and clinical complexity were observed via the direct pathways.

#### 3.3.6. Comparison across Models

The least parsimonious models were the models including the latent variables morbidity burden and patient complexity¸ which included the highest number of variables (Table 3). Among those with a directly measured variable, the comorbidity discordance model was the most parsimonious. The AIC was larger for women than for men, suggesting a better fit of the models for men.

## 4. Discussion

In this study, we examined the association between patient-reported patient safety and a range of comorbidity measures derived from the literature. For women, and as hypothesized, a consistent association between higher levels of the constructs and lower levels of patient safety was observed indirectly via an increased number of visits (patient complexity), directly (number of conditions and comorbidity discordance) or both (morbidity burden). While the findings for men were consistent with these observations for the indirect pathway (polypharmacy, morbidity burden and patient complexity), we also found an unexpected and simultaneous yet remarkably consistent direct association with better (rather than worse) patient safety for these constructs.

### 4.1. Discussion of Main Findings and Comparison with the Previous Literature

The reasons for the observed gender difference are not fully clear. Although there did not seem to be any differences in number of conditions, there were gender differences in the proportion of each condition—in particular, the most frequent conditions among women were more frequently mutually discordant than for men (just 29% of those conditions with a prevalence higher than 15% among women were concordant while the proportion was 67% for men (all cardiometabolic)). The differential associations between the raw number of conditions (and medications) and patient safety could be attributed to differential proportions of comorbidity discordance due to differential epidemiological profiles linked to gender. However, this would also explain no differential effect being observed for the GSEM for comorbidity discordance. This would also, at least in part, explain that the differential effect was also observed for the morbidity burden and clinical complexity, which included both the number of conditions and medications as well as comorbidity discordance in their construction, as well as other variables. However, the effect did not seem to be attenuated by the inclusion of a larger number of additional variables in the clinical complexity index (five) compared to the morbidity index (two).

It is also conceivable that the differences may be related to different frequencies in the presence of other conditions not included in the self-report of our study and, also, by different patterns of association (clusters) with implications for management that may not be fully captured by our approach to measuring comorbidity discordance (we note that we were only able to define and measure concordance a priori based on conditions, rather than a posteriori, based on actual concordance between medications). They could also be an expression of larger gender healthcare inequalities that have already been established in a large number of other areas of healthcare. There is no reason to presume that these are necessarily mutually exclusive explanations.

The “comorbidity discordance” model was the most parsimonious for both women and men. Since this model was one of the three not including a latent variable as a multimorbidity construct, it would suggest that models for “multimorbidity” and “polypharmacy” would be underfit, whereas the more complex models with the latent variables “morbidity burden” and “patient complexity” (which included all the previous constructs) would be overfit.

A previous comprehensive systematic review identified that different types of multimorbidities resulted in different risks of adverse patient safety outcomes. In particular, a physical–mental comorbidity was associated with an increased risk of both active and precursors of safety incidents whereas physical comorbidity was associated only with a smaller risk active safety incidents [18]. Our findings would be consistent with these observations that comorbidity discordance (physical–mental comorbidity being an example) is associated with worse levels of patient safety. No stratification was done for gender.

A previous study with 1190 patients registered in 45 practices did not show any association between multimorbidity and polypharmacy and selected PREOS-PC scores in a regression model with patient and practice activation as independent variables and adjusted for a range of clinical and sociodemographic characteristics, but not gender, as it was not statistically significantly associated with the dependent variables in bivariate analyses [43].

Similarly, a recent study aiming to identify high-need patients with multimorbidities concluded that people who frequently contact the general practice use general practice out-of-office services or have unplanned admissions that are largely distinct high-need subgroups but did identify gender-specific differences [44].

### 4.2. Strengths and Limitations

This study has several strengths. The population included is largely representative of the broad primary care reference population, patient safety was measured using a validated tool and appropriate analytical approaches were implemented to model the relevant associations. A number of limitations also need to be considered. First, its cross-sectional design limits the inferences about causality. We cannot rule out that, in some cases, the safety events experienced could have led to increased multimorbidity or polypharmacy. There is good evidence that safety incidents are relatively common in the primary care setting and are of significant importance when considered as a whole, but most of them do not result in serious harm [3]. This was also the case in our study, in which the severity of harm reported by the study participants was low (as evidenced by the high scores on the harm scales; Table 2). Therefore, this potential reversed causality is unlikely to have played a major role in this study. Future studies should consider using a longitudinal design to overcome this limitation. Second, this is a secondary analysis of the baseline data of two trials. The sample size was therefore limited and may have contributed to a lack of convergence of the multilevel GSEM analyses. Further, the available data on self-reported morbidity were also limited based on the protocol for the original study. The inclusion of additional diseases in the modelling would have allowed to obtain a more comprehensive description of the morbidity status of the individual. Although this is likely to have had the biggest impact on the multimorbidity and discordant comorbidity and, to some degree, on the latent indexes that we constructed based on them, it must be noted that these indexes relied on additional variables, thereby attenuating the impact of either the multimorbidity -or discordant multimorbidity on the latent variables. Thirdly, the multimorbidity latent variables morbidity burden and patient complexity were developed using variables that the models for multimorbidity, polypharmacy and discordance were adjusted for. Although it would not have been appropriate to adjust for these variables in the multimorbidity latent variables model, it may be argued that the resulting models may benefit from further adjustment.

### 4.3. Implications for Research, Practice, Policy

Our study suggests that it is feasible to operationalize a range of different multimorbidity constructs presented in the literature. Further research implementing both similar and alternative operationalisations—in particular, including the locus of control and continuity of care as additional variables in the construction of the clinical complexity index [45,46]—are necessary for further validation of the approach and for exploring the associations in other contexts. The comorbidity discordance appears as a promising instrument in establishing the mechanisms by which multimorbidity is associated with worse healthcare outcomes [47]. Reanalysis of the existing data with a comorbidity discordant approach may provide an efficient approach to shedding light on the mechanisms by which previous associations with more blunt approaches (count of conditions) have been reported [48].

Further research is needed to replicate the observed gender-specific differences and, if confirmed, to elucidate their nature and operating mechanisms.

Our latent class analysis of PREOS-PC present with an opportunity for the consideration of the overall scores that may facilitate the interpretation of patient feedback and its communication to health professionals.

For women, particularly those with discordant comorbidity, the observed detrimental effects of multimorbidity and related constructs on patient safety make it necessary that efforts in the development, implementation and evaluation of the safe process of care prioritize these vulnerable patients. In the absence of standards of care based on robust evidence for people with multimorbidities [16], in particular, patient safety appears as a key focus in the provision of care.

## 5. Conclusions

In this study, we observed that, in women, an increased number of conditions, number of medications, comorbidity discordance, morbidity burden and patient complexity are associated with worse patient safety (either through direct associations, through indirect associations mediated by number of visits or both). In men, the pattern of associations is more complex: although increased polypharmacy, morbidity burden and patient complexity are associated with worse safety through an indirect pathway (mediated by an increased number of visits), at the same time, these three multimorbidity constructs are unexpectedly associated with better safety through a direct pathway.

Women with discordant multimorbidities appear as a relevant target group for the development of interventions aimed at improving patient safety in primary care. Further research—particularly, qualitative research—is needed for clarifying the complex associations among men.

## Figures and Tables

**Table 1:** Sociodemographic and clinical characteristics of the participants.

	Women (*n* = 3059; 64%)	Men (*n* = 1723; 36%)	Total (*n* = 4782)
Age			
Mean (SD)	51.12 (18)	54.06 (19)	52.1 (19)
<18	56 (2%)	60 (3%)	116 (2%)
18–29	346 (11%)	160 (9%)	506 (11%)
30–44	747 (24%)	315 (18%)	1062 (22%)
45–64	1097 (36%)	581 (34%)	1678 (35%)
≥65	811 (27%)	607 (35%)	1418 (30%)
Educational level			
University studies	597 (19%)	244 (14%)	835 (16%)
Other qualifications	1744 (57%)	1075 (62%)	2816 (59%)
No qualifications	724 (24%)	404 (23%)	1128 (24%)
Country of origin			
Spain	2618 (86%)	1546 (90%)	4164 (87%)
Other country (European Union)	122 (4%)	56 (3%)	178 (4%)
Other country (Non-European Union)	319 (10%)	121 (7%)	440 (9%)
Occupational status			
Working	1532 (50%)	804 (47%)	2336 (49%)
Unemployed	342 (11%)	87 (5%)	429 (9%)
Retired	862 (28%)	710 (41%)	1572 (33%)
Other (student, volunteering, etc.)	323 (11%)	122 (7%)	445 (9%)
Visits to PHC centre in the previous 12 months			
1–5	1672 (55%)	993 (58%)	2665 (56%)
6–10	767 (25%)	388 (23%)	1155 (24%)
11–20	395 (13%)	230 (13%)	625 (13%)
>20	225 (7%)	112 (7%)	337 (7%)
Health status			
Very good	366 (12%)	237 (14%)	603 (13%)
Good	1420 (46%)	891 (52%)	2311 (48%)
Fair	999 (33%)	472 (27%)	1471 (31%)
Bad	208 (7%)	96 (6%)	304 (6%)
Very bad	66 (2%)	27 (2%)	93 (2%)
Number of long-term conditions			
Mean (SD; range)	2.20 (2.17; 0–16)	2.13 (1.95; 0–10)	2.17 (2.09; 0–16)
0	816 (27%)	441 (26%)	1257 (26%)
1	628 (21%)	331 (19%)	959 (20%)
2 to 3	865 (28%)	638 (37%)	1433 (30%)
>3	750 (25%)	383 (22%)	1131 (24%)
Long-term conditions			
Hypertension	815 (27%)	611 (35%)	1426 (30%)
Hypercholesterolemia	684 (22%)	510 (30%)	1195 (25%)
Diabetes	309 (10%)	333 (19%)	642 (13%)
Asthma or bronchitis or emphysema	322 (11%)	178 (10%)	500 (10%)
Long-term heart problem	250 (8%)	284 (16%)	534 (11%)
Stomach ulcer	134 (4%)	58 (3%)	192 (4%)
Allergy	597 (20%)	244 (14%)	841 (18%)
Depression	523 (17%)	151 (9%)	674 (14%)
Other mental health problems	187 (6%)	91 (5%)	278 (6%)
Migraine/headaches	578 (19%)	107 (6%)	685 (14%)
Blood circulation problems	587 (19%)	219 (13%)	806 (17%)
Hernia	274 (9%)	220 (13%)	494 (10%)
Arthrosis and rheumatic problems	921 (30%)	349 (20%)	1270 (27%)
Osteoporosis	305 (10%)	25 (1%)	330 (7%)
Menstruation-related problems	232 (8%)	-	232 (5%)
Prostate-related problems	-	287 (17%)	287 (6%)
Number of medications			
Mean (SD; range)	2.25 (2.94; 0–30)	2.61 (3.15; 0–27)	2.38 (3.02; 0–30)
0	1129 (38%)	560 (33%)	1689 (36%)
1	472 (16%)	253 (15%)	725 (16%)
2–4	1038 (35%)	607 (36%)	1645 (35%)
5–10	282 (9%)	216 (13%)	498 (11%)
>10	67 (2%)	40 (2%)	107 (2%)

**Table 2:** Patient-reported patient safety with the PREOS-PC questionnaire.

	Women		Men		Total	
	Mean (SD) Score	Score Range (Min–Max)	Mean (SD) Score	Score Range (Min–Max)	Mean (SD) Score	Score Range (Min–Max)
Patient activation	38.13 (36.93)	6.25–100	39.99 (37.85)	0–100	38.80 (37.27)	0–100
Team activation	79.81 (20.22)	6.25–100	84.01 (18.32)	18.75–100	81.32 (19.66)	6.25–100
Experiences of safety events	91.81 (13.33)	0–100	93.51 (12.25)	0–100	92.42 (12.98)	0–100
Harm (severity)	96.43 (11.86)	0–100	96.83 (11.75)	0–100	96.58 (11.81)	0–100
Harm (needs)	95.98 (12.61)	0–100	96.97 (10.99)	0–100	96.34 (12.06)	0–100
Overall rating of patient safety	83.51 (16.50)	0–100	84.82 (15.55)	0–100	83.98 (16.18)	0–100

**Table 3:** Associations between the different measures of multimorbidity, patient safety and number of visits by sex in the final generalized structural equation models *.

	Women (β (95% CI))				Men (β (95% CI))			
	Indirect Pathway		Direct Pathway	AIC	Indirect Pathway		Direct Pathway	AIC
	MM to Visits	Visits to PS	MM to PS		MM to Visits	Visits to PS	MM to PS	
Number of conditions ⱡ	0.15 (0.13 to 0.16) *	−0.25 (−0.24 to 0.74)	−0.83 (−1.08 to −0.57) *	129,086.9	0.16 (0.14 to 0.18) *	−0.09 (−0.65 to 0.47)	0.15 (−0.17 to 0.48)	71,554.63
Number of medications ⱡ	0.12 (0.10 to 0.13) *	−0.22 (−0.73 to 0.29)	−0.09 (−0.28 to 0.10)	126,845.7	0.12 (0.11 to 0.14) *	−0.54 (−1.08 to −0.01) *	0.21 (0.03 to 0.39) *	69,863.42
Comorbidity discordance ⱡ				69,106.07				39,370.94
Completely concordant (ref.)	-	−0.18 (−0.46 to 0.82)	-		-	−0.12 (−0.69 to 0.46)	-	
Predominantly concordant	0.32 (0.10 to 0.54) *		−3.67 (−6.44 to −0.90) *		0.30 (0.09 to 0.52) *		0.18 (−1.78 to 2.15)	
Predominantly discordant	0.02 (−0.15 to 0.20)		−3.34 (−5.53 to −1.16) *		0.19 (0.03 to 0.35) *		−0.43 (−1.88 to 1.03)	
Completely discordant	−0.30 (−0.49 to −0.12) *		−0.89 (−3.27 to 1.49)		−0.18 (−0.36 to −0.01) *		−0.52 (−2.20 to 1.16)	
Morbidity burden ¶	0.34 (0.30 to 0.37) *	−0.88 (−1.36 to −0.40) *	−0.67 (−1.17 to −0.18) *	191,973	0.36 (0.31 to 0.41) *	−0.96 (−1.42 to −0.51) *	0.79 (0.32 to 1.26) *	107,340
Patient Complexity †	0.36 (0.32 to 0.40) *	−0.79 (−1.27 to −0.31) *	−0.11 (−0.61 to 0.39)	211,882.2	0.38 (0.33 to 0.43) *	−1.00 (−1.46 to −0.53) *	0.87 (0.38 to 1.36) *	117,935

β: unstandardized coefficients, CI: confidence interval, AIC: Akaike information criterion, MM: multimorbidity, PS: patient safety, Visits: number of visits to the primary care centre during the last 12 months and ref: reference category. ⱡ adjusted by age, educational attainment and self-reported health status. * Statistically significant associations (*p* < 0.05) are noted with an asterisk (*).¶ adjusted by educational attainment (age and self-reported health status already included in the development of the “Morbidity burden” latent variable). † not adjusted (age, educational attainment and self-reported health status already included in the development of the “Patient Complexity” latent variable). Dark grey cells: path consistent with the direction of a priori hypotheses, white cells: path not consistent or opposed to the a priori hypotheses and pale grey cells: path in the opposite direction of the a priori hypotheses.

## Data Availability

Ignacio Ricci-Cabello was the grantor of the data in this study.

## Supplementary Materials

The following are available online at https://www.mdpi.com/article/10.3390/jcm10081782/s1: Online Appendix 1. Bivariate and adjusted associations between the patient safety, number of visits and multimorbidity constructs by gender.

## Appendix A. Description of the Latent Variables

**Table A1:** Description of the morbidity burden latent variables: pairwise correlations between the proposed indicators and the latent variables and loadings in the confirmatory factor analysis.

Morbidity burden	Pairwise Correlations		Confirmatory Factor Analysis Loadings (95% CI)	
	Women	Men	Women	Men
Number of conditions	0.99 *	0.99 *	0.96 (0.95 to 0.97)	0.98 (0.97 to 0.99)
Discordance of conditions	0.92 *	0.89 *	0.89 (0.88 to 0.90)	0.88 (0.86 to 0.89)
Number of medications	0.66 *	0.68 *	0.64 (0.62 to 0.66)	0.67 (0.64 to 0.70)
Age	0.58 *	0.59 *	0.57 (0.54 to 0.60)	0.58 (0.55 to 0.62)
Self-reported health status ^a^	0.47 *	0.38 *	0.46 (0.43 to 0.49)	0.38 (0.34 to 0.42)

* Statistically significant correlations (*p* < 0.001). ^a^ Higher scores indicative of worse self-reported health status.

**Table A2:** Description of the patient complexity latent variables: pairwise correlations between each of the selected indicators for patient complexity and the patient complexity latent variable and loadings in the confirmatory factor analysis.

Patient Complexity	Pairwise Correlations		Confirmatory Factor Analysis (Loadings (95% CI))	
	Women	Men	Women	Men
Number of conditions	0.98 *	0.99 *	0.96 (0.95 to 0.96)	0.97 (0.97 to 0.98)
Concordance of the conditions	0.92 *	0.90 *	0.90 (0.89 to 0.90)	0.88 (0.87 to 0.89)
Number of medications	0.67 *	0.69 *	0.65 (0.63 to 0.67)	0.67 (0.65 to 0.70)
Age	0.59 *	0.60 *	0.58 (0.55 to 0.60)	0.59 (0.56 to 0.63)
Self-reported health status ^a^	0.47 *	0.39 *	0.47 (0.44 to 0.50)	0.38 (0.34 to 0.42)
Educational attainment ^b^	−0.38 *	−0.24 *	0.38 (0.33 to 0.40)	0.23 (0.18 to 0.28)
Occupational status ^c^	0.26 *	0.33 *	0.25 (0.22 to 0.29)	0.33 (0.28 to 0.37)
Country of origin ^d^	−0.12 *	−0.14 *	0.12 (0.08 to 0.16)	0.14 (0.09 to 0.18)

* Statistically significant correlations (*p* < 0.001). ^a^ Higher scores indicative of a worse self-reported health status, ^b^ higher scores indicative of higher educational attainment, ^c^ higher scores indicative of a worse occupational status and ^d^ higher scores indicative of belonging to a non-EU country.

**Table A3:** Description of the patient safety latent variables: pairwise correlations between scales and the latent variables and loadings in the confirmatory factor analysis.

Patient Safety	Pairwise Correlations		Confirmatory Factor Analysis Loadings (95% CI)	
	Women	Men	Women	Men
Team activation	0.60 *	0.55 *	0.52 (0.48 to 0.55)	0.46 (0.41 to 0.51)
Experiences of safety events	0.93 *	0.90 *	0.79 (0.76 to 0.84)	0.75 (0.70 to 0.81)
Harm-severity	0.56 *	0.59 *	0.48 (0.44 to 0.52)	0.50 (0.45 to 0.54)
Harm-needs	0.55 *	0.57 *	0.47 (0.43 to 0.51)	0.48 (0.43 to 0.53)
Overall rating of patient safety	0.65 *	0.67 *	0.56 (0.52 to 0.60)	0.56 (0.51 to 0.61)

* Statistically significant correlations (*p* < 0.001).
