# Supplementary material for: Assessing the Impact of Multi-Morbidity and Related Constructs on Patient Reported Safety in Primary Care: Generalized Structural Equation Modelling of Observational Data
Source: J Clin Med. 2021 Apr 20;10(8):1782. doi: 10.3390/jcm10081782 (PMC8073542; doi:10.3390/jcm10081782)
Supplement: Supplementary file 1 [file jcm-10-01782-s001.zip › jcm-1075744-supplementary.pdf]

Online Appendix 1. Bivariate and adjusted associations between patient safety, number of visits, and multimorbidity constructs, by gender

Table 1a. Bivariate and adjusted associations between patient safety overall score and the multimorbidity measures, by gender: regression coefficients and 95% Confidence Intervals.

	Patient Safety (simple model)				Patient Safety (multilevel model)			
	**Men[c]**	**Men[a]**	**Women[c]**	**Women[a]**	**Men[c]**	**Men[a]**	**Women[c]**	**Women[a]**
Number conditions ǂ	0.25 (0.08 to 0.42)	0.08 (-0.13 to 0.29)	-0.24 (-0.39 to -0.10)	-0.58 (-0.76 to -0.41)	0.24 (0.06 to 0.41)	0.09 (-0.12 to 0.30)	-0.24 (-0.39 to -0.10)	-0.56 (-0.74 to -0.38)
Number prescriptions ǂ	0.18 (0.07 to 0.29)	0.12 (0.01 to 0.25)	0.01 (-0.10 to 0.11)	-0.05 (-0.18 to 0.08)	0.19 (0.08 to 0.30)	0.14 (0.02 to 0.27)	-0.03 (-0.14 to 0.08)	-0.07 (-0.20 to 0.06)
Comorbidity discordance ǂ	-0.44 (-0.85 to -0.03)	-0.16 (-0.56 to 0.24)	-0.92 (-1.43 to -0.41)	-0.03 (-0.55 to 0.49)	-0.45 (-0.86 to -0.04)	-0.17 (-0.57 to 0.23)	-0.89 (-1.40 to -0.38)	-0.04 (-0.56 to 0.47)
Morbidity burden ¶	0.43 (0.08 to 0.78)	0.70 (0.35 to 1.05)	-0.42 (-0.74 to -0.10)	-0.55 (-0.90 to -0.20)	0.39 (0.04 to 0.74)	0.65 (0.30 to 1.00)	-0.44 (-0.76 to -0.12)	-0.58 (-0.93 to -0.24)
Patient Complexity †	0.73 (0.36 to 1.09)	0.71 (0.35 to 1.07)	-0.08 (-0.43 to 0.27)	-0.10 (-0.44 to 0.24)	0.67 (0.31 to 1.03)	0.66 (0.30 to 1.01)	-0.12 (-0.47 to 0.23)	-0.13 (-0.47 to 0.21)

ǂ adjusted by age, educational attainment and self-reported health status
c, crude (unadjusted) associations; a, adjusted associations.
¶ adjusted by educational attainment (age and self-reported health status already included in the model for the development of the "Morbidity burden" index)
† not adjusted (age, educational attainment and self-reported health status already included in the model for the development of the "Patient Complexity" index)

Table 1b. Bivariate and adjusted associations between number of visits and multimorbidity constructs, by gender: regression coefficients and 95% Confidence Intervals.

	**Number of visits (simple model)**				**Number of visits (multilevel model)**			
	**Men[c]**	**Men[a]**	**Women[c]**	**Women[a]**	**Men[c]**	**Men[a]**	**Women[c]**	**Women[a]**
Number conditions ƚ	0.16<br>(0.14 to 0.18)	0.10<br>(0.07 to 0.13)	0.15<br>(0.13 to 0.16)	0.09<br>(0.07 to 0.10)	0.16<br>(0.14 to 0.18)	0.10<br>(0.08 to 0.13)	0.14<br>(0.13 to 0.16)	0.09<br>(0.07 to 0.10)
Number prescriptions ƚ	0.12<br>(0.11 to 0.14)	0.10<br>(0.08 to 0.12)	0.12<br>(0.10 to 0.13)	0.07<br>(0.06 to 0.09)	0.12<br>(0.11 to 0.14)	0.10<br>(0.09 to 0.12)	0.12<br>(0.10 to 0.13)	0.07<br>(0.06 to 0.09)
Comorbidity discordance ƚ	-0.05<br>(-0.11 to 0.01)	-0.02<br>(-0.08 to 0.04)	-0.15<br>(-0.21 to -0.10)	-0.09<br>(-0.15 to -0.04)	-0.05<br>(-0.11 to 0.01)	-0.02<br>(-0.08 to 0.03)	-0.16<br>(-0.21 to -0.10)	-0.10<br>(-0.15 to -0.04)
Morbidity burden ¶	0.36<br>(0.32 to 0.40)	0.34<br>(0.30 to 0.38)	0.34<br>(0.30 to 0.37)	0.30<br>(0.26 to 0.33)	0.36<br>(0.31 to 0.40)	0.28<br>(0.23 to 0.33)	0.33<br>(0.30 to 0.37)	0.21<br>(0.17 to 0.24)
Patient Complexity †	0.38<br>(0.34 to 0.43)	0.38<br>(0.34 to 0.43)	0.36<br>(0.32 to 0.39)	0.36<br>(0.32 to 0.39)	0.38<br>(0.34 to 0.42)	0.38<br>(0.34 to 0.42)	0.36<br>(0.33 to 0.39)	0.36<br>(0.33 to 0.39)

ƚ adjusted by age, educational attainment and self-reported health status
c, crude (unadjusted) associations; a, adjusted associations.
¶ adjusted by educational attainment (age and self-reported health status already included in the model for the development of the "Morbidity burden" index)
† not adjusted (age, educational attainment and self-reported health status already included in the model as for the development of the "Patient Complexity" index)

Table 1c. Bivariate and adjusted associations between number of visits and patient safety, by gender: regression coefficients and 95% Confidence Intervals.

	**Number of visits (simple model)**				**Number of visits (multilevel model)**			
	**Men[c]**	**Men[a]**	**Women[c]**	**Women[a]**	**Men[c]**	**Men[a]**	**Women[c]**	**Women[a]**
Patient Safety	-0.24<br>(-0.60 to 0.11)	-0.08<br>(-0.45 to 0.28)	-0.41<br>(-0.74 to -0.07)	-0.03<br>(-0.38 to 0.32)	-0.27<br>(-0.63 to 0.08)	-0.09<br>(-0.45 to 0.28)	-0.48<br>(-0.81 to -0.14)	-0.10<br>(-0.44 to 0.25)

c, crude (unadjusted) associations; a, adjusted associations (adjusted by age, educational attainment and self-reported health status).
